# Myélome multiple survenant au cours d'une Fièvre Méditerranéenne Familiale

**DOI:** 10.11604/pamj.2013.15.123.1434

**Published:** 2013-08-07

**Authors:** Bouomrani Salem, Farah Afef, Bouassida Nadia, Ayadi Nabil, Bahloul Zouhir, Béji Maher

**Affiliations:** 1 Hôpital Militaire de Gabes 6000-Tunisie; 2 Service de Médecine Interne, Hôpital Hédi Chaker, Sfax, Tunisie

**Keywords:** Fièvre méditerranéenne familiale, maladie périodique, myélome multiple, plasmocytome, cancer, Familial Mediterranean Fever, periodic disease, multiple Myeloma, plasmocytoma, cancer

## Abstract

L'objectif de ce travail est de rapporter une observation particulière de myélome multiple survenant au cours d'une maladie périodique. Il s'agit d'un patient tunisien de 53 ans suivi depuis le jeune âge pour maladie périodique dont le diagnostic était confirmé par l’étude génétique montrant l'homozygotie pour la mutation M694V du gène MEFV, fut admis pour exploration d'une douleur avec tuméfaction fessière droite récente. Les explorations biologiques et radiologiques ont permis de retenir le diagnostic d'un myélome multiple de type IgA à chaînes légères kappa stade III B, associé à une volumineuse localisation plasmocytaire très agressive de l'aile iliaque droite envahissant les structures musculaires avoisinantes. Notre observation, qui à notre connaissance est la deuxième signalant une telle association, se distingue par sa survenue brutale, sa progression rapide et le caractère très agressif de l'hémopathie.

## Introduction

La maladie périodique, mieux encore appelée Fièvre Méditerranéenne Familiale (FMF) est une maladie inflammatoire chronique à évolution paroxystique. Elle fut décrite pour la première fois en 1945 [1] et caractérisée génétiquement en 1992 [2, 3]. C'est une affection à caractère familiale et à transmission principalement autosomique récessive. Elle est particulièrement fréquente chez les arméniens, les turques, les juifs sépharades et les arabes du moyen orient [1, 4]. Son substratum génétique est une mutation ponctuelle du gène «MEFV» situé sur le bras court du chromosome 16 [1, 4, 5]. La transcription de ce gène aboutit à la production d'une protéine baptisée «pyrine» ou «marénostrine» qui intervient dans la régulation de la réponse inflammatoire, en particulier leucocytaire et l'apoptose cellulaire [4, 5].

Sur le plan clinique, la FMF se caractérise par des accès paroxystiques douloureux et fébriles abdominaux, thoraciques et articulaires auxquels peuvent s'associer diverses manifestations systémiques: cardiaques, neurologiques, cutanées, urogénitales et hématopoïétiques. Son diagnostic clinique est basé sur des critères diagnostiques dont les plus utilisés sont ceux de Livneh [6] et sa confirmation reste du domaine de la cytogénétique par la mise en évidence de mutations du gène «MEFV» dont il existe plus d'une vingtaine. Les plus fréquentes de ces mutations sont: M694V, M694I, V726A et E148Q [4, 7]. Sa complication majeure est l'amylose AA qui domine le pronostic [1, 4].

L'association FMF et myélome multiple (MM) est très exceptionnelle. Elle n’était rapportée qu'une seule fois auparavant [8]. A travers cette deuxième observation, nous essayons de discuter les mécanismes possibles de cette association ainsi que ces éventuelles particularités cliniques.

## Patient et observation

Homme tunisien de 53 ans connu ayant la maladie périodique sous colchicine 1 mg/j depuis 1994. Le diagnostic de FMF était retenu devant la recrudescence depuis le jeune âge de crises paroxystiques douloureuses et fébriles abdominales associées à des arthrites inflammatoires séronégatives des poignets, chevilles et genoux et des signes cutanés: érythème érysipétatoïde et placards celluo dermiques douloureux siégeant au niveau du tronc et des jambes avec un syndrome inflammatoire biologique marqué et une sensibilité nette des symptômes à la colchicine. Le diagnostic fut confirmé par une étude génétique montant une homozygotie pour la mutation M694V du gène MEFV.

**Figure 1:**
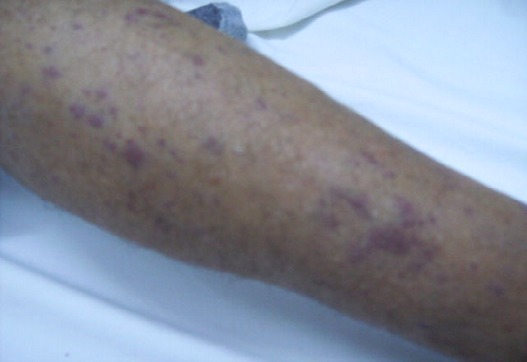
Lésions cutanées peu infiltrées et érythémato-violacées papuleuses des jambes accompagnant les poussées de FMF chez notre patient

Il fut hospitalisé dans notre service en Mars 2010 pour douleur et tuméfaction fessière droite constatées depuis cinq mois et s'aggravant rapidement. L'examen somatique trouve un état général altéré et un patient très algique, une éruption cutanée des deux membres inférieurs faite de petits placards cellulo dermiques violacés (Figure 1) et une masse sous cutanée de consistance molle, de forme ovalaire, faisant 6/4 cm, sensible et située au niveau de la fesse droite en regard de l’épine iliaque supéro externe sans signes inflammatoires en regard. La mobilisation active et passive de la hanche droite était douloureuse avec une nette limitation du secteur de mobilité surtout l'abduction: 5° et la rotation interne: 10°. Il n'y avait pas d'adénopathies palpables ni d'hépato splénomégalie. Le bilan biologique montrait une anémie normochrome normocytaire à 7.4 g/l sans thrombopénie ni leucopénie, une vitesse de sédimentation élevée à 120 mmH1, une protéine C réactive à 24 mg/l, une hyper protidémie à 129 g/l avec une protéinurie de 24h à 0,3 g, une calcémie à 2.64 mmol/l, une insuffisance rénale avec créatinine à 220 µmol/l et une hyper uricémie à 638 µmol/l. l’électrophorèse des protéines plasmatiques objectivait une hyper gammaglobulinémie monoclonale à 51.6 g/l avec répression des autres protéines. L'immunoélectrophorèse des protéines sanguines isolait une gammapathie monoclonale type IgA à chaînes légère Kappa et celle des protéines urinaires montrait des chaînes légères kappa monoclonales libres et liées.

**Figure 2:**
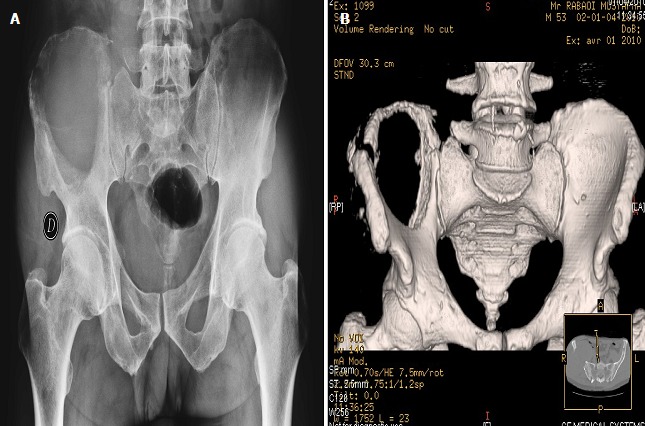
Radiographie standard du bassin de face (A) et TDM du bassin en reconstitution tridimensionnelle sans injection de produit de contraste (B): grande lacune au niveau de l'os iliaque droit avec lyse corticale

**Figure 3:**
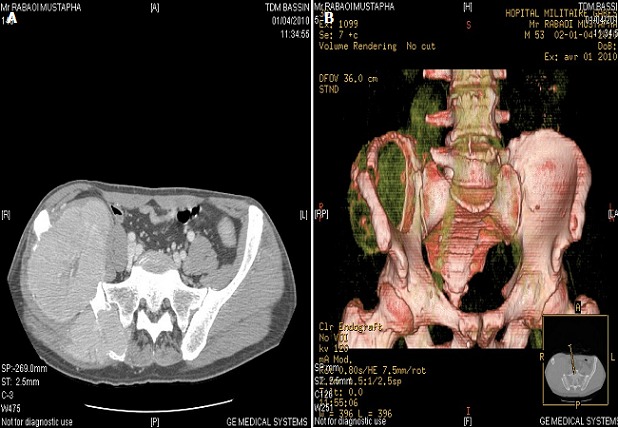
TDM du bassin en coupe axiale non injectée et (A) en reconstruction tridimensionnelle avec injection de produit de contraste; (B) en temps artériel: processus tumoral agressif centré sur l'os iliaque droit avec rupture de la corticale osseuse et envahissement des structures adjacentes. Cette tumeur a la même densité que les gros vaisseaux témoignant de son caractère hyper vascularisé

La radiographie standard de bassin montrait une importante lésion lytique de l'aile iliaque droite (Figure 2). Le scanner X thoraco-abdomino-pelvien trouvait une volumineuse masse tissulaire hypodense lytique avec rupture de la corticale, centrée sur l'os iliaque droit et qui arrivait jusqu'en regard de l'articulation sacro iliaque et envahissait en avant le chef iliaque et en arrière les muscles para vertébraux et fessiers droits (Figure 2, Figure 3). Ils s'y associaient d'autres lésions lytiques à l'emporte-pièce des corps vertébraux de la première et cinquième vertèbre lombaires et de l'os iliaque gauche ainsi qu'un niveau de l'arc moyen de la 7^ème^ cote droite avec fracture pathologique en regard.

La ponction sternale confirmait le diagnostic de myélome multiple en montrant l'infiltration de la moelle par 30% de plasmocytes dystrophiques. Les radiographies standard des os long et du crâne ne montrent pas de lésions géodiques. Il n'y avait pas de signes de compression neurologique à l'imagerie par résonance magnétique nucléaire médullaire.

Au terme de ce bilan, le diagnostic d'un myélome multiple à IgA stade III B avec une localisation plasmocytaire tumorale agressive de l'os iliaque droit et des tissus mous adjacents fut retenu et le patient était soumis à un traitement d'induction séquentiel par dexaméthasone^®^ et thalidomide^®^ en préparation à une auto greffe de cellules souches médullaires.

## Discussion

L'association maladies inflammatoires chroniques-néoplasies est décrite par plusieurs auteurs et avec plusieurs pathologies. L'inflammation chronique des différents tissus et le dérèglement immunitaire qui caractérisent ces affections sont les principaux facteurs incriminés dans cette carcinogenèse; Ils s'y ajoutent la susceptibilité génétique et l'usage des thérapeutiques immunosuppressives [9].

Au cours de la FMF, seules quelques observations sporadiques d'association à des tumeurs solides ou hémopathies sont rapportées. Il s'agit principalement de mésothélium péritonéal [10–13]. Plus rarement sont décrits le mésothélium pleural [14] et vésical [15], le carcinome broncho alvéolaire [16], le carcinome rénal [17], le cancer colorectal [18], les leucémies myéloïdes [5], le lymphome gastrique [19] et la tumeur carcinoïde [20].

Le caractère favorisant de greffe néoplasique que présente la maladie périodique parait fort probable; particulièrement pour le mésothélium péritonéal dont l'incidence au cours de cette affection est significativement plus importante que celle dans la population générale dans les pays concernés [10]. Ces constatations restent cependant controversées [14].

Cette hypothèse se trouve aussi renforcée par la coexistence de plusieurs néoplasies chez le même patient atteint de FMF comme l'illustrait l'observation de Kirkpantur et al: association d'un adénocarcinome rénal et d'un carcinome urétéral invasif et peu différencié chez un patient atteint de maladie périodique compliquée d'amylose AA [17].

L'association d'une FMF à un MM n'est rapportée qu'une seule fois dans la littérature mondiale [8].

Le mécanisme pathogénique de ces associations reste encore incertain. Plusieurs hypothèses sont émises; en particulier:

**L'inflammation chronique:** le rôle favorisant des cytokines pro inflammatoires dans la pathogénie des gammapathies monoclonales est démontré [21] et le rôle particulier de l'IL-6 dans le développement des MM a été signalé [22]. L'excès de production de l'IL-6 secondaire au déficit de C5-inhibitor caractéristique de la FMF pourrait stimuler, en tant que facteur de croissance paracrine, la prolifération maligne d'un clone de plasmocytes exprimant le récepteur à cette cytokine [8]. Cette hypothèse se trouve réconfortée par deux constatations: une hypergammaglobulinémie pourtant sur les IgA, IgG, IgM et IgD retrouvée respectivement dans 23%, 17%, 13% et 13% des FMF [4] et la mise en évidence d'un comportement anormal similaire des cellules lymphocytaires ainsi que leurs noyaux et nucléoles au cours des leucémies et des maladies périodiques [23] expliquant le pouvoir oncogène potentiel de ces affections. -Un effet carcinogène direct du gène MEFV est aussi suspecté, en particulier pour le développement des hémopathies malignes: Tidow et al. ont objectivé l'expression de ce gène mutant au niveau de 8/11 lignées cellulaires néoplasiques de sujets ayant une leucémie myéloïde et au niveau d'une lignée cellulaire/11 au cours des leucémies lymphoïdes [5]. Au cours de cette même étude, ce gène ainsi que son transcrit mutant (ARNm) ont été retrouvés exprimés par les cellules de quatre lignées néoplasiques coliques/11: SW480, SW837, HuTu80 et SW1417 et trois lignées cellulaires de cancer prostatique: LNCal, PC-3 et DU145. Cette implication directe du gène MEFV dans l'oncogenèse reste encore controversée, d'autant plus qu'aucune différence significative n'a été notée quant à son expression prostatique et colique entre sujets cancéreux et sains [5].

Une étude récente faite par Oktenlic et al. appuie cette hypothèse en montrant une expression fréquente du gène MEFV mutant chez les sujets présentant une hémopathie malignes en dehors de toute histoire personnelle ou familiale de maladie périodique! cette association était plus nette pour les syndromes myélodysplasiques: 66,6%, la polyglobulie vraie: 33,3% et les leucémies aigues myéloïdes: 28,6% [24].

**Rôle probable de la pyrine**: il a été démontré récemment que la pyrine contient un domaine de «mort» nommé PYD pour « Pyrin Domain» impliqué dans la régulation de la mort cellulaire programmée ou apoptose [4]. Il serait donc possible q'une modification de ce domaine, structurale ou fonctionnelle, secondaire à la mutation pourrait être à l'origine d'une prolifération cellulaire non contrôlée’ En effet le rôle réprimant de la pyrine sur l'apoptose leucocytaire st signalé [1]. Cette prolifération cellulaire toucherait plus particulièrement les cellules hématopoïétiques; cellules où l'expression du gène MEFV est prédominante, aboutissant à l'hémopathie et secondairement les autres tissus (colon, prostate.) aboutissant aux tumeurs solides.

## Conclusion

L'association entre FMF et cancer est loin d’être un simple hasard. Les mécanismes physiopathologiques favorisant une telle association ne sont pas encore bien élucidés. Au centre des facteurs plausibles de l'oncogenèse au cours de la FMF se trouve l'inflammation chronique, l'hypercytokinémie, la mutation du gène MEFV et sa protéine qui en résulte. Une surveillance particulière clinique et biologique se trouve ainsi justifiée chez les patients avec la maladie périodique à fin de dépister à temps les néoplasies.
